# A Case Report of Intraoral Injection of Botulinum Toxin A for Trigeminal Neuralgia: A Rare but Safe Intervention

**DOI:** 10.7759/cureus.80456

**Published:** 2025-03-12

**Authors:** Richard F Radlberger, Stefan Leis

**Affiliations:** 1 Department of Neurology, Christian‑Doppler University Hospital, Paracelsus Medical University, Centre for Cognitive Neuroscience, EpiCARE, Salzburg, AUT

**Keywords:** botulinum toxin, case report, facial pain, intraoral, trigeminal neuralgia

## Abstract

Although off-label, according to the recommendations from guidelines, subcutaneous injections with botulinum toxin A in a follow-the-pain pattern are increasingly being used in trigeminal neuralgia. Subsequently, we report on an elderly woman with trigeminal neuralgia who responded well to intraoral therapy in addition to the subcutaneous injections in the dermal area of the affected trigeminal branch.

## Introduction

Trigeminal neuralgia (TN) with its shooting, electric shock-like attacks is a pain disorder with an enormous impact on quality of life, which can even develop into a life-threatening condition due to impaired food intake should chewing result in severe pain. The diagnostic criteria, according to the third edition of the International Classification of Headache Disorders (ICHD-3) [1], refer to recurrent paroxysms of unilateral facial pain in the distribution(s) of one or more divisions of the trigeminal nerve, with no radiation beyond. The pain characteristics have to be lasting from a fraction of a second to two minutes, with severe intensity and electric shock-like, shooting, stabbing, or sharp in quality. Some attacks may be, or appear to be, spontaneous, but there must be a history or finding of pain provoked by innocuous stimuli. Furthermore, they must not be better accounted for by another International Classification of Headache Disorders (ICHD-3) diagnosis. While classic TN is caused by a neurovascular conflict, secondary TN is caused by an underlying disease, and idiopathic TN has no abnormalities on magnetic resonance imaging (MRI). Available treatment options include systemic medication and localized treatment strategies. The subcutaneous injection of onabotulinum toxin A into the affected area leads to a significant pain reduction [2], and botulinum toxin (BoNTX) is now also recommended as a second or third choice treatment option in several guidelines [3-5]. Nevertheless, as far as we know, intraoral submucosal injections are mostly done by dentists or oral surgeons and are only rarely performed by neurologists [6,7]. We present a case report to encourage neurologists to perform intraoral injections of BoNTX for TN.

## Case presentation

**Figure 1:**
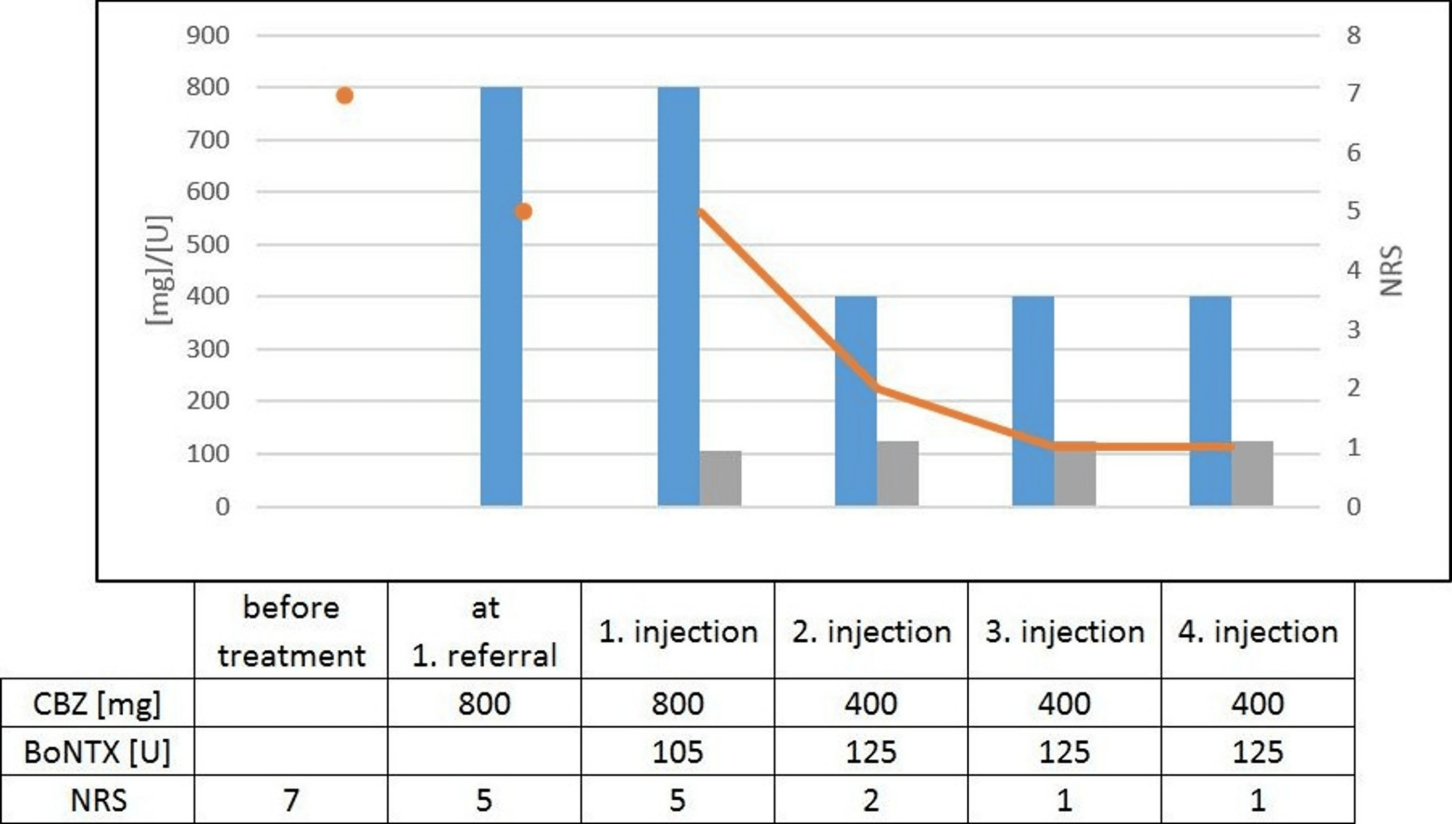
Course of medication and NRS Shown is the dosage of the drug used in milligrams or units (left axis scaling) in relation to the specified pain level measured by the NRS (right axis scaling), with the blue bars representing the dosage of carbamazepine and the grey ones representing the dosage of BoNTX. The orange dots, as well as the line diagram of the same colour, indicate the course of the NRS. CBZ: carbamazepine, mg: milligram, NRS: numerical rating scale, BoNTX: botulinum toxin, U: units

A woman in her seventies was referred by her general practitioner to the outpatient clinic of our department of neurology. She described a facial pain in the area of the second branch of the trigeminal nerve on her right side that started about seven years earlier. The pain was sharp and shooting repeatedly for parts of seconds and was interrupted by pain-free intervals. The frequency of the attacks varied and could sometimes be triggered by touching the affected area or when chewing. Magnetic resonance imaging showed no neurovascular conflict or any symptomatic causes for TN. Consequently, the diagnosis of an idiopathic TN was made. Treatment with carbamazepine was associated with nausea, inappetence, and obstipation, which led to an insufficient maximum tolerated dose of 800 milligrams daily. The oral medication resulted in a reduction of two points on the numerical rating scale (NRS) from seven to five but hardly any reduction of the attack frequency. We discussed different treatment options, especially rotation to oxcarbazepine or add-on therapy with gabapentin or pregabalin. Because of the side effects of carbamazepine, she was dismissive of another systemic therapy and asked for a focal treatment. She was informed about possible side effects, especially asymmetry in her facial expressions, and we performed the first injection with BoNTX in a chessboard pattern of one to one and a half centimeters in the affected area. Each injection consisted of five units of incobotulinum toxin A, summing up to 105 units. At the next follow-up visit 14 weeks later, she described a marked reduction of three points on the NRS from five to two even though she lowered the daily intake of carbamazepine to 400 milligrams daily with no side effects due to the systemic drug. In addition to the alleviation of pain, she also reported a decrease in frequency of around thirty percent. Figure 1 shows the indicated values of the NRS and the applied dose of the individual drugs.

**Figure 2:**
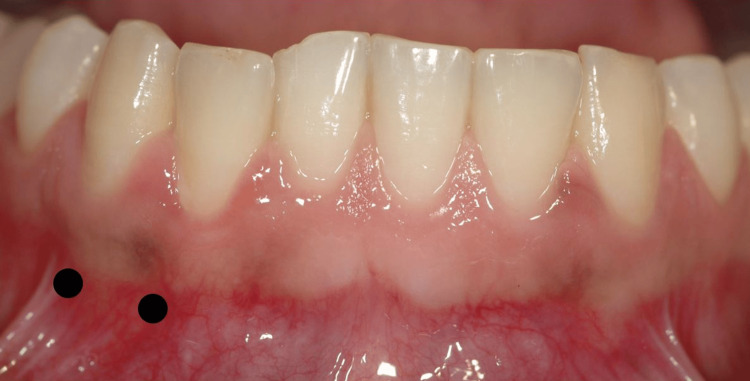
Injection sites The image, taken by Mohamed Hamze, has been taken from the public domain of Wikimedia Commons and shows the injection sites (black dots) in the area around the canines.

Interestingly, she then showed us a trigger zone at the canine tooth that she had not noticed until then. Accordingly, we tried an injection at the junction of the gingiva and the mucosa, as shown in Figure 2, with 10 units at each flank of the tooth, resulting in a further reduction in pain intensity to one to two on the NRS. The frequency of attacks was hardly altered, but it was noteworthy that the triggering was completely absent now when eating.

## Discussion

Summing up the initial case, it is worth mentioning that the onset was roughly seven years earlier than her first admission to our outpatient clinic. At this time, treatment with carbamazepine was established with a tolerated maximum dosage of 800 milligrams daily. Due to the patient’s reservation, a medication rotation was subsequently rejected even though she negated other treatment attempts. It would have been interesting if a rotation to oxcarbazepine would have performed better regarding side effects. As recommended in various guidelines [3-5], gabapentinoids should be discussed either as an alternative to the first-line treatment or as add-on therapy. Combination therapy might lead to dose savings and a reduction in side effects.

Out of the anticonvulsive drugs, lamotrigine, topiramate, levetiracetam, lacosamide, phenytoin, valproic acid, and eslicarbazepine have additionally been described as effective in maintenance therapy [3,8,9]. Baclofen is described as comparable in its responses and side effect rate when used as monotherapy [10]. When used in combination, carbamazepine and gabapentin appear to perform better [11].

The use of these drugs is often limited by their systemic side effects. Local infiltration with BoNTX has been proposed as an alternative [12]. BoNTX probably exerts its antinociceptive effect by inhibiting the release of neuropeptides from primary nociceptive afferents [13]. Four placebo-controlled randomized studies, all carried out in China, showed a favorable effect of subcutaneous or submucosal injection of up to 100 units of onabotulinum toxin A in the treatment of TN and were summarized in a meta-analysis [2].

Considering a shared decision-making process, we have chosen a focal treatment strategy with BoNTX as second-line therapy to prevent systemic side effects. The administered dose in our case was determined solely by the size of the affected area. Accordingly, we were slightly above the described upper limit for the first injection [2].

Keeping in mind the literature on intraoral injections with BoNTX, we did find some reports of dentists using incobotulinum [6] or onabotulinum [7] toxin A and consequently performed the injection. After our patient reported further improvement, as described above, we surprisingly noticed that it is a rather uncommon practice to neurologists and, if addressed, frequently linked to reservations. Considering its safety profile, the mentioned publication [7] only described a temporary mucosal dryness in the injected intraoral area that affected food intake only on a minimal scale. Upon inquiry, our patient negated mucosal dryness in the area of the injection and discomfort when eating. For example, after the initial injection, there was asymmetry in the facial muscles, which was described as not bothersome, even though the additional injections in the trigger zone totalled 125 units incobotulinum toxin A per visit. Meanwhile, a constant pain reduction over roughly one year was realized by treatment sessions with intraoral injections carried out at intervals of around 14 weeks.

## Conclusions

To our knowledge, there are no relevant side effects published on the intraoral use of BoNTX, as described above. Hence, when a patient under treatment with BoNTX due to trigeminal neuralgia points to an intraoral trigger zone, additional local injections should be considered. Subsequent research has to further clarify the role of this treatment option in comparison to common strategies.

## References

[1] (2018). Headache Classification Committee of the International Headache Society (IHS) The International Classification of Headache Disorders, 3rd edition. Cephalalgia.

[2] Morra ME, Elgebaly A, Elmaraezy A (2016). Therapeutic efficacy and safety of botulinum toxin A therapy in trigeminal neuralgia: a systematic review and meta-analysis of randomized controlled trials. J Headache Pain.

[3] Ruscheweyh R, Gierthmühlen J, Hedderich DM, Goßrau G, Leis S (2024). Trigeminal neuralgia: drug therapy : the new German guideline [Article in French]. Schmerz.

[4] Chong MS, Bahra A, Zakrzewska JM (2023). Guidelines for the management of trigeminal neuralgia. Cleve Clin J Med.

[5] Bendtsen L, Zakrzewska JM, Abbott J (2019). European Academy of Neurology guideline on trigeminal neuralgia. Eur J Neurol.

[6] Wojtecki L, Maierhofer O, Albrecht P (2021). Intraoral alveolar submucosal injections of Incobotulinumtoxin A: Relief of therapy-refractory trigeminal neuropathy after tooth extraction. eNeurologicalSci.

[7] Herrero Babiloni A, Kapos FP, Nixdorf DR (2016). Intraoral administration of botulinum toxin for trigeminal neuropathic pain. Oral Surg Oral Med Oral Pathol Oral Radiol.

[8] Lambru G, Zakrzewska J, Matharu M (2021). Trigeminal neuralgia: a practical guide. Pract Neurol.

[9] Gambeta E, Chichorro JG, Zamponi GW (2020). Trigeminal neuralgia: an overview from pathophysiology to pharmacological treatments. Mol Pain.

[10] Sangamesh NC, Bajoria AA, Mishra S, Behera S, Sahoo SK, Bal PK (2024). Comparative assessment of the effectiveness of carbamazepine and baclofen in the management of trigeminal neuralgia. J Pharm Bioallied Sci.

[11] Murugesan I, T N U, Ramalingam K (2024). A retrospective analysis of medical management strategies for trigeminal neuralgia: an institutional review. Cureus.

[12] Micheli F, Scorticati MC, Raina G (2002). Beneficial effects of botulinum toxin type a for patients with painful tic convulsif. Clin Neuropharmacol.

[13] Aoki KR (2005). Review of a proposed mechanism for the antinociceptive action of botulinum toxin type A. Neurotoxicology.

